# Distinct extracellular architectures shape mechanical persistence in GPVI and CLEC-2 receptor assemblies

**DOI:** 10.1371/journal.pcbi.1014884

**Published:** 2026-10-13

**Authors:** Yao Zhang, Yue Cheng, Han Ni Soe, Zhipeng Xu

**Affiliations:** 1 School of Mathematics and Statistics, Nantong University, Nantong, China; 2 Laboratory of Biostatistics and Computational Medicine, Nantong University, Nantong, China; University of Maryland School of Pharmacy, UNITED STATES OF AMERICA

## Abstract

Glycoprotein VI (GPVI) and C-type lectin-like receptor 2 (CLEC-2) are platelet-activating receptors whose extracellular organization contributes to receptor association and signaling during thrombus formation, yet how alternative receptor architectures influence mechanical persistence remains unclear. We used molecular dynamics to compare a GPVI dimer based on 2GI7, a domain-swapped GPVI assembly derived from 7NMU, and a crystallographic CLEC-2 contact using the same pulling protocol and constant-force conditions. During the first 5 nm of imposed separation, reconstructed GPVI required less force and mechanical work than CLEC-2 and domain-swapped GPVI. Under constant-force loading from 400 to 600 pN, reconstructed GPVI also reached the same extension endpoint markedly earlier than the domain-swapped assembly, with passage times differing by more than an order of magnitude under several conditions. Extended pulling to approximately 10 nm produced complete loss of interreceptor heavy-atom contacts in reconstructed GPVI while local peptide and disulfide connectivity remained intact. In contrast, the domain-swapped preparation retained resistance over a larger part of the separation pathway and showed substantially greater mechanical persistence. These results show that alternative extracellular arrangements of the same receptor are not mechanically equivalent and that uncertainty in GPVI architecture can translate into large differences in force transmission and persistence under load. Such architecture-dependent persistence may influence the stability of extracellular receptor associations during thrombus formation and thereby affect the mechanical lifetime of receptor clusters under load.

## Author summary

Platelets form clots by recognizing molecules around them and organizing surface receptors into signaling assemblies. Two important platelet receptors, glycoprotein VI (GPVI) and CLEC-2, can experience mechanical forces as platelets interact with their surroundings during thrombus formation. GPVI has been observed in different extracellular arrangements, but it is not clear whether these structures respond differently when mechanical forces tend to pull associated receptors apart.

Using molecular dynamics simulations, we compared two GPVI extracellular arrangements with a CLEC-2 receptor pair under the same loading protocol. The GPVI preparation separated relatively rapidly, whereas the domain-swapped GPVI arrangement resisted separation for substantially longer and accumulated much greater mechanical work. These results show that alternative extracellular arrangements of the same platelet receptor can exhibit markedly different mechanical persistence under force. Such architecture-dependent persistence may influence how long extracellular receptor associations and receptor clusters remain mechanically intact during thrombus formation.

## 1 Introduction

Glycoprotein VI (GPVI) couples recognition of exposed collagen to platelet activation through an extracellular region containing two immunoglobulin domains, making the organization of these domains relevant to both ligand engagement and receptor association [1,2]. Crystal structures of GPVI bound to collagen peptides locate the binding surface on the D1 β-sheet. The spacing of accessible collagen motifs further constrains how neighboring GPVI molecules can engage the same triple helix [2]. Fluorescence measurements also support a mixture of monomers and dimers, including evidence for endogenous dimers on platelet membranes, connecting this structural question to receptor organization on cells [3].

The extracellular arrangements available to GPVI include the dimer deposited as 2GI7 and an assembly with exchange between the D2 domains deposited as 7NMU, whose extended hinge changes how neighboring receptor molecules are arranged [1,4]. A subsequently resolved monomer retained a D1 and D2 orientation comparable to that in a dimer, showing that this domain orientation alone is compatible with more than one association state of the extracellular receptor [5]. More recently, an Affimer that selectively binds dimeric GPVI identified an additional recognition site on D2, providing further evidence that GPVI dimerization is associated with distinct extracellular structural features [6].

CLEC-2 provides a biologically related comparison because it is another platelet activating receptor, but its compact extracellular lectin fold differs markedly from the paired immunoglobulin domains of GPVI [7]. Structures with podoplanin and rhodocytin show that these chemically different ligands engage the side face of CLEC-2 through shared acidic and arginine interactions together with additional interactions specific to each ligand [8]. More recent cellular experiments and models of receptor clustering show that ligand valency, receptor abundance, receptor dimerization and recruitment of intracellular tandem SH2 domain proteins cooperate in receptor clustering, placing extracellular association within a larger process that depends on both molecular structure and cellular organization [9].

Direct observations of GPVI and CLEC-2 movement further distinguish encounters between receptors from associations stabilized by multivalent ligands, a distinction that is important when comparing molecular persistence across the two receptor families [10]. At low expression in CHO-K1 cells lacking Syk, both receptors moved mainly as monomers, whereas addition of multivalent ligands promoted association and immobilization, with CLEC-2 associations persisting longer than those of GPVI [10]. These findings motivate further study of how an associated extracellular pair responds to a separating force, while ligand geometry, membrane diffusion, and intracellular recruitment remain separate contributors to cellular persistence.

The mechanics of such a pair depend on how force is applied as well as on the interactions holding its constituent receptors together, as anticipated by Bell’s treatment of reversible molecular bonds in specific cell adhesion [11]. Evans and Ritchie subsequently showed that the strength of a molecular adhesion bond depends on the rate of force application and that different loading regimes expose different aspects of the underlying dissociation process [12]. For extracellular receptor assemblies, this distinction motivates separate measurements of the largest opposing force, the work delivered during imposed displacement and the time required to reach a specified extension under sustained force, accompanied by measurements of receptor deformation and interface separation.

Here we investigate how the extracellular arrangement and covalent connectivity of prepared GPVI assemblies accompany differences in mechanical resistance, comparing a 2GI7 model with reconstructed internal loops with the 7NMU assembly and a selected crystallographic CLEC-2 pair. Molecular dynamics simulations follow the response of associated receptor pairs to moving restraints and sustained forces, comparing their mechanical resistance and the time required to reach a common extension threshold. By considering force, delivered work, extension, interreceptor contacts and deformation of each receptor together, we ask whether separation proceeds mainly through opening of the interface or through structural changes within the loaded receptor groups.

## 2 Materials and methods

### 2.1 GPVI structural models

The deposited 2GI7 structure contains unresolved segments in chain B at residues 99–107 and 130–137, while chain A begins at deposited residue 2 [1]. These gaps interrupt the peptide continuity of chain B and therefore require completion before the covalent connectivity of the receptor pair can be represented consistently in the mechanical calculations.

To obtain a continuous-chain GPVI model, the missing residues in chain B were repaired while retaining the experimentally resolved coordinates. The completed model contains 182 residues in chain A and 184 in chain B, giving 366 residues in total. The inserted loops connect the resolved segments of chain B and were accepted only when the intended residue coverage and plausible peptide geometry were maintained at each reconstructed boundary. The resulting preparation contains 181 peptide bonds in chain A and 183 in chain B, together with two intrachain disulfide bonds, Cys28–Cys68 and Cys114–Cys160, in each receptor. The two receptor groups contain 2803 and 2827 atoms, respectively, including 1412 and 1425 heavy atoms, and these complete groups were used for alignment, contact analysis, and mechanical loading.

The alternative GPVI preparation was based on the domain-swapped structure 7NMU, which contains four covalent units designated A, C, B, and D, with A + C and B + D forming the two receptor groups [4]. Each longer fragment contains a Cys32–Cys72 disulfide, whereas the close sulfur pairs A118–D164 and C164–B118 remain nonbonded. Thus, the 7NMU preparation differs from the continuous-chain 2GI7 model not only in extracellular arrangement but also in the covalent connections through which force is transmitted across each receptor group. Both GPVI preparations were subjected to equilibrium molecular dynamics and subsequent mechanical loading simulations for direct comparison.

### 2.2 CLEC-2 structural model

The CLEC-2 preparation is a selected crystallographic contact generated from PDB entry 2C6U by a symmetry operation, providing an associated extracellular pair with a fold distinct from that of GPVI [7]. The deposited biological assembly contains one receptor chain, and the selected pair is evaluated here as a candidate interface under the same mechanical conditions used for the GPVI comparison. The CLEC-2 system was simulated for 300 ns in five independent runs, and the final configurations were used to initiate the mechanical loading calculations. The subsequent moving-restraint simulations were run for 50 ns at 0.1 nm ns^–1^, corresponding to 5 nm of imposed displacement along the loading direction.

CLEC-2 and GPVI were compared under the same moving-restraint conditions, using identical simulation time, pulling velocity, and harmonic stiffness. Force and mechanical work were therefore evaluated over the same 5 nm displacement. Longer GPVI pulling simulations were analyzed separately, while constant-force simulations were compared using the same extension threshold and the corresponding event-time uncertainty.

### 2.3 Equilibrium sampling

All molecular dynamics simulations were performed using GROMACS 2023.2 [13]. Equilibrium simulations of the 2GI7-based GPVI dimer were performed in five independent runs, each extending to 300 ns. The final configuration from each run was used as a starting structure for the mechanical calculations. The same starting configurations were retained across the constant-force conditions, allowing the response to different applied forces to be compared consistently. Structural changes during the late equilibrium stage were evaluated from 200 to 300 ns at 1 ns intervals relative to the initial configuration of each trajectory.

The complete dimer and individual receptors were aligned separately for structural analysis. Simulations used AMBER99SB-ILDN and TIP3P water with neutralization and nominal 0.15 M sodium chloride, a 2 fs integration step, hydrogen-bond constraints, particle-mesh electrostatics, and nominal 1.2 nm electrostatic and van der Waals cutoffs [13–17]. Temperature was maintained at 310 K by velocity rescaling with a 0.1 ps coupling time, and equilibrium pressure used isotropic stochastic cell rescaling at 1 bar with a 2 ps coupling time [18,19].

For the matched CLEC-2 loading starts, each solvated system contained two protein chains, 28391–30694 water molecules, 83–90 Na^+^ ions, and 83–90 Cl^-^ ions, giving 89263–96186 total atoms in boxes spanning 6.85–7.53×6.80–7.53×18.09–18.60 nm. For the repaired GPVI starts, each solvated system contained two protein chains, 51818–66203 water molecules, 151–187 Na^+^ ions, and 159–195 Cl^-^ ions, giving 161394–204621 total atoms in boxes spanning 7.06–8.11×7.26–8.26×30.71–31.44 nm. The CLEC-2 setup used 50 ps of NVT equilibration followed by 100 ps of NPT equilibration before the mechanical starts, whereas the reconstructed GPVI setup used 100 ps of NVT equilibration followed by 1 ns of NPT equilibration before equilibrium production and subsequent reboxing for loading.

Mechanical loading started from the final equilibrated configurations after transfer to elongated simulation boxes. Isotropic stochastic cell rescaling was retained during the moving-restraint simulations, whereas the constant-force simulations were performed at fixed volume. The GPVI systems contained 5630 protein atoms, with total system sizes ranging from 161394 to 204621 atoms depending on the solvent volume and box dimensions required for each starting configuration.

### 2.4 Mechanical loading

Figure 1 illustrates the receptor geometry and the direction of mechanical loading. The loading coordinate *q* is defined as the projection of the displacement between the mass-weighted centers of the two receptor groups onto a fixed separation axis. Equal and opposite forces are applied to the two receptor groups along this axis. For the moving-restraint simulations, the harmonic restraint moves from its initial position at a constant velocity, and the difference between the restraint position and the instantaneous coordinate determines the signed separating force.

**Fig 1:**
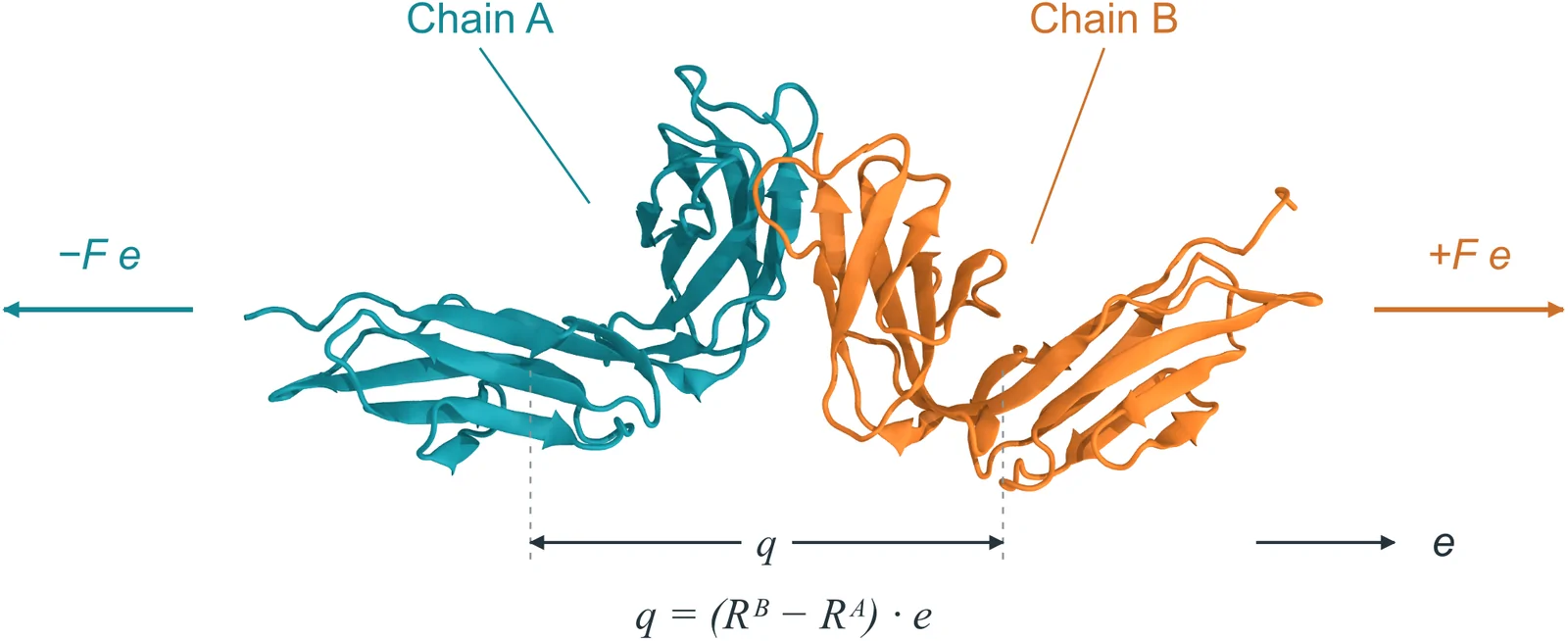
Molecular geometry and mechanical loading of the 2GI7 GPVI dimer. Chain A is shown in teal and chain B in orange. Schematic force arrows indicate the loading direction, and the directional center separation defines the coordinate used to follow receptor separation.

Mechanical work was calculated by integrating the separating force over the prescribed restraint displacement. For 7NMU, both covalent fragments belonging to each receptor group were treated together when defining the group center and applying the force, so that the loading coordinate consistently followed the motion of the complete receptor groups [13].

Moving-restraint simulations used a harmonic stiffness of 1000 kJ mol^–1^ nm^–2^ and a pulling velocity of 0.1 nm ns^–1^. The primary comparison between CLEC-2 and GPVI was restricted to 50 ns, corresponding to the same imposed displacement of 5 nm, so that force and mechanical work were compared under identical loading conditions. At this displacement, CLEC-2 had lost the receptor–receptor association, whereas the GPVI assemblies remained associated. The GPVI simulations were therefore extended to 100 ns, corresponding to 10 nm of imposed displacement, to determine how further separation proceeded and whether interreceptor contacts were eventually lost. Maximum force was defined as the largest 100 ps running mean of the force magnitude, and mechanical work was calculated by trapezoidal integration of the force over the imposed displacement.

Constant-force simulations were performed at 400, 500, and 600 pN for each of the five starting configurations, with a maximum simulation time of 50 ns for each condition. The 2GI7-based GPVI simulations used a directional extension target of + 10 nm, whereas the additional 7NMU simulations used a target of + 5 nm, both measured relative to the corresponding initial coordinate. Comparisons between the GPVI preparations were therefore made at the common 5 nm extension threshold, while the longer 10 nm extension of the 2GI7-based GPVI simulations was analyzed separately.

Interreceptor contacts were defined using a 0.6 nm heavy-atom cutoff, and receptor deformation was assessed from Cα RMSD, radius of gyration, end-to-end distance, and extension along the loading direction. Peptide and disulfide distances were also monitored to assess chain continuity during mechanical loading.

### 2.5 Survival analysis of separation times

Survival analysis was used to estimate a force-dependent separation timescale that can be related to experimental measurements of receptor persistence. The threshold time was defined as the first time the directional separation reached the specified extension from its initial value. Trajectories that did not reach the threshold within the observation window were treated as right censored, while bounded crossing times retained their interval uncertainty. The survival function describes the probability that the threshold has not yet been reached at time *t*, and its integral provides the corresponding mean timescale without assigning artificial separation times to incomplete trajectories [20–23].

For a separation threshold *d*, the survival probability at constant force *F* is defined as


$$\begin{array}{c} S_{d}(t|F)=\mathrm{Pr}(T_{d}>t|F), \end{array}$$ (1)

where $T_{d}$ is the first time the directional separation reaches *d* relative to its initial value. Each crossing was represented by an interval $\left(L_{i},U_{i}\right]$ containing the event time. Trajectories that remained below the threshold at the end of the 50 ns observation window were treated as right censored rather than assigned a separation time [20,21]. The mean threshold time is given by the area under the survival curve,


$$\begin{array}{c} \tau_{d}(F)=\int_{0}^{\infty }S_{d}(t|F)\;dt. \end{array}$$ (2)

Because the simulations have a finite observation window *H* = 50 ns, we also calculated the restricted mean time,


$$\begin{array}{c} \mu_{H}(F)=\int_{0}^{H}S_{d}(t|F)\;dt, \end{array}$$ (3)

which uses only the observed time range [22,23]. Lower and upper event-time bounds were retained when calculating the survival curves and restricted mean times to account for uncertainty in the exact crossing time.

### 2.6 Lifetime extrapolation

Force-dependent separation rates were described using a Bell-type relation [11]. Assuming exponential survival at each applied force, the model parameters were estimated by maximum likelihood using the observed threshold-time intervals and right-censored trajectories. The fitted rate *k*(*F*) was converted to the corresponding mean separation time as


$$\begin{array}{c} \tau (F)=\frac{1}{k(F)}. \end{array}$$

The force dependence of *k*(*F*) was then used to estimate the separation timescale at lower forces, including the extrapolated zero-force value. Uncertainty was assessed by resampling the starting configurations within each receptor model.

### 2.7 Statistical analysis

The analyses used five independent equilibrium trajectories for each model. Force and mechanical work over the common 5 nm displacement were compared using exact two-sided permutation tests, with Holm correction for multiple comparisons [24].

Constant-force comparisons between the 2GI7-based and 7NMU GPVI models retained the observed crossing-time intervals and right-censored trajectories. Preparation sensitivity was assessed by comparing the independent 2GI7 ensembles over the same 5 nm displacement using permutation tests for force and work, with resampling used to estimate uncertainty.

## 3 Results

### 3.1 Repaired chains

The repaired GPVI model contains continuous peptide chains across both internal chain B gaps and two disulfide bonds within each receptor, establishing the covalent connections present during the subsequent mechanical calculations. Figure 2 shows the initial structures of the 2GI7-based GPVI dimer, the four-fragment 7NMU model, and the CLEC-2 contact, highlighting their different extracellular arrangements before loading. The same receptor-group definitions were used throughout the analysis for force, work, directional extension, and interreceptor contact measurements.

**Fig 2:**
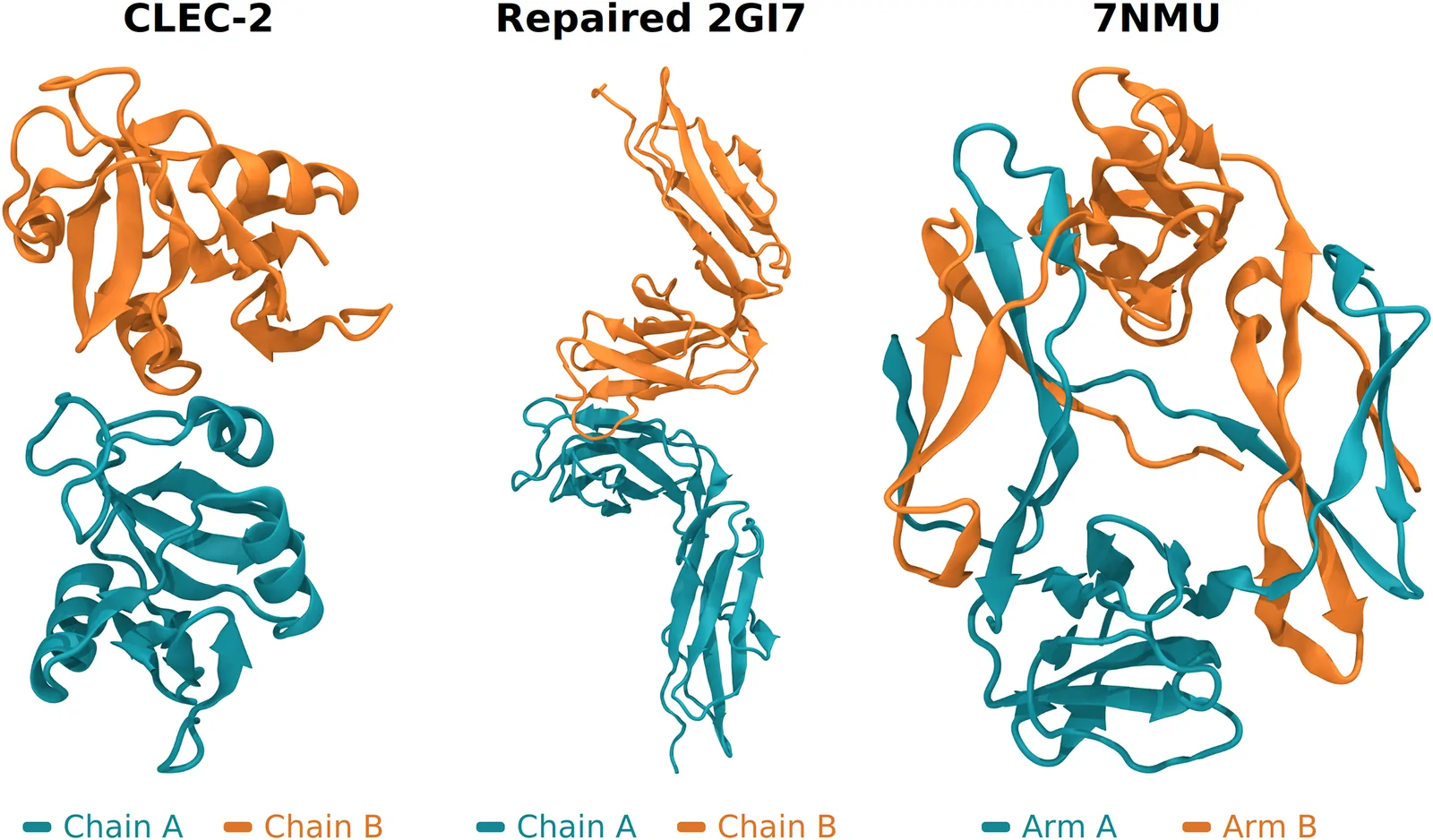
Initial receptor configurations shown as ribbon structures. The panels show the CLEC-2 reference, repaired 2GI7, and the longer displacement 7NMU preparation. Colors distinguish the two force groups; in 7NMU each group contains two covalent fragments. The structures show the extracellular arrangements used for the loading calculations.

During 200–300 ns, mean dimer Cα RMSD ranged from 0.458 to 0.888 nm, while the corresponding values after separate alignment of chain A and chain B ranged from 0.222 to 0.264 nm and from 0.321 to 0.419 nm, respectively. The trajectory with the largest dimer displacement showed less pronounced displacement after individual receptor alignment, indicating that relative motion between the receptors contributed to the overall structural change. Interchain heavy-atom contacts within 0.6 nm were retained throughout the analyzed interval.

Figure 3A, 3B and 3D summarize the late equilibrium behavior through mean trajectories and shaded uncertainty bands across five independent simulations, while panel C compares the trajectory-specific temporal mean Cα RMSDs of the separately aligned chains and the complete dimer. The minimum interchain heavy-atom pair count within 0.6 nm was 483, while the minimum-image interchain distance ranged from 0.2443 to 0.2916 nm, consistent with persistent close association throughout the sampled interval. The higher dimer RMSD relative to the separately aligned chains suggests that changes in the relative arrangement of the two receptors contribute more strongly than deformation within each receptor. These associated structures were subsequently used as the starting points for mechanical loading.

**Fig 3:**
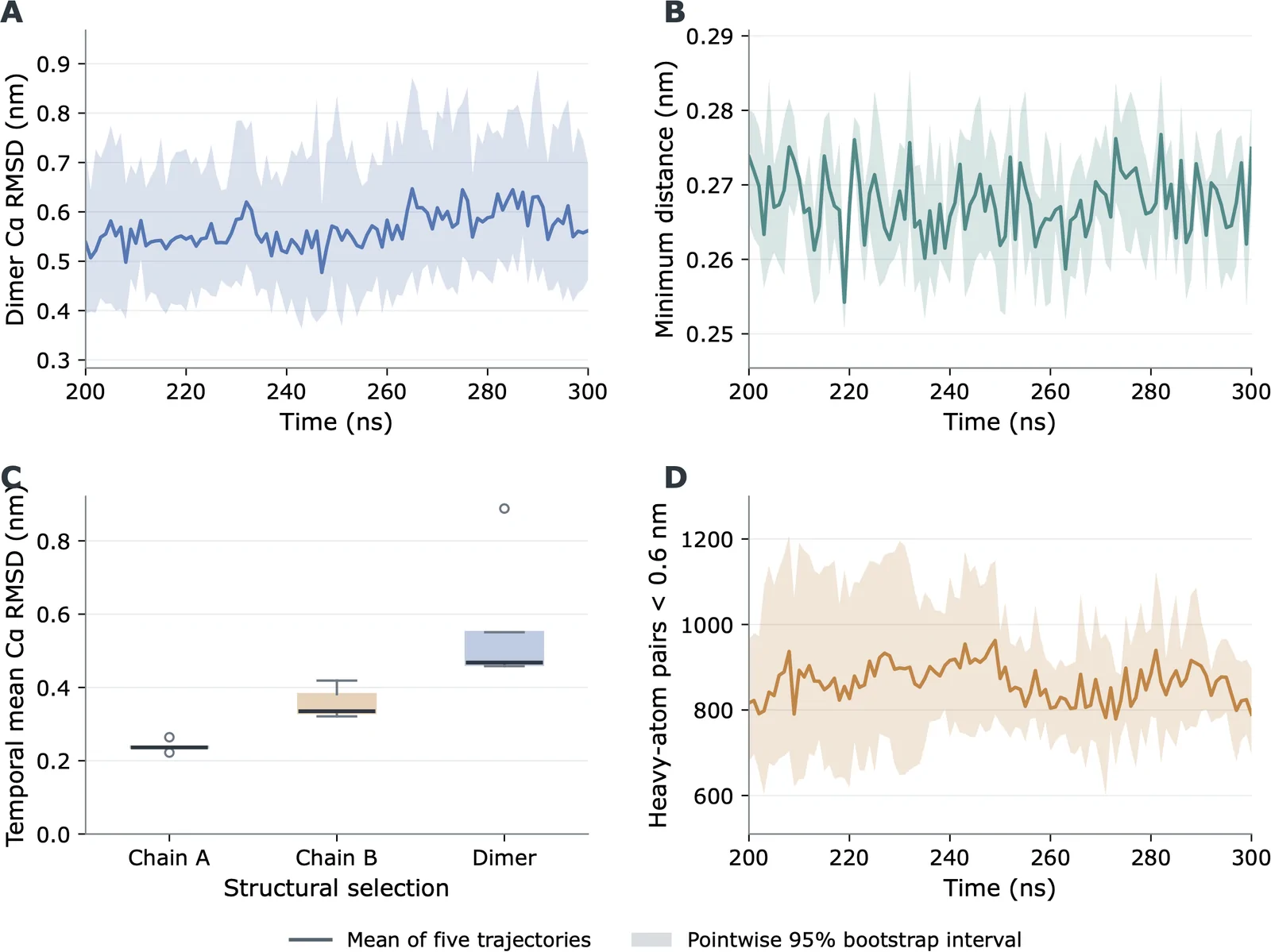
Late equilibrium behavior of the 2GI7-based GPVI dimer. Panels **A** and **B** show dimer Cα RMSD and the minimum interchain heavy-atom distance, respectively, while panel **D** shows the number of interchain heavy-atom contacts within 0.6 nm. Solid curves indicate the mean behavior and shaded regions show the corresponding uncertainty. Panel **C** summarizes the temporal mean Cα RMSD of chain A, chain B, and the complete dimer.

### 3.2 Mechanical response under matched loading

The force and accumulated-work trajectories over the common 5 nm displacement are shown in Fig 4, with the corresponding summary statistics listed in Table 1. Figure 5A shows a clear separation of repaired 2GI7 from the other two preparations over the initial 5 nm of restraint travel, with median maximum 100 ps mean forces of 674.6 pN for CLEC-2, 354.6 pN for repaired 2GI7, and 576.8 pN for 7NMU. All observed maximum mean forces for CLEC-2 and 7NMU were higher than those for the 2GI7-based GPVI dimer, with *p* = 0.00794 for both comparisons. The CLEC-2 and 7NMU distributions overlap and their adjusted probability is 0.222, showing a less distinct force separation than either comparison with repaired 2GI7.

**Fig 4:**
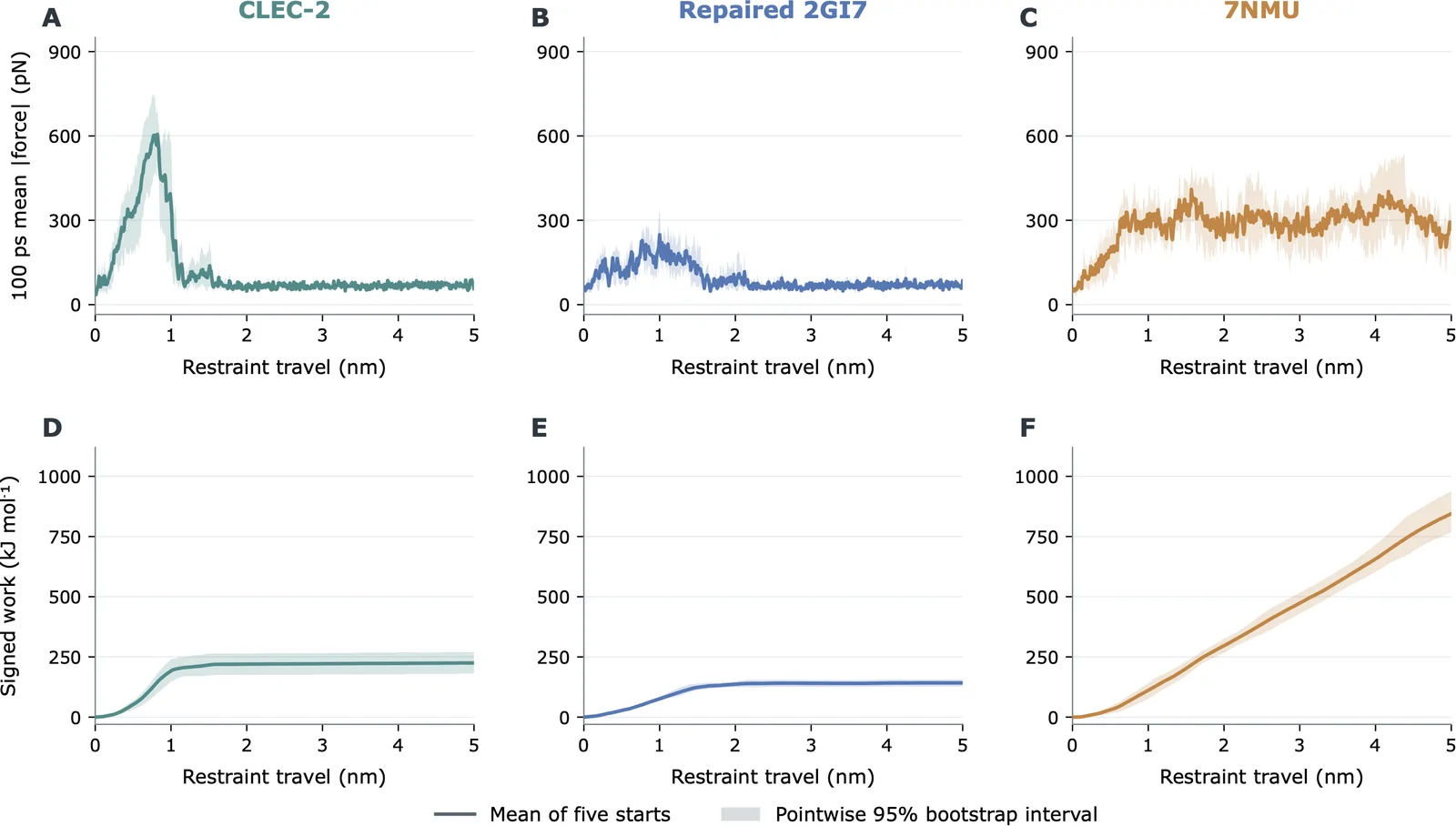
Force and accumulated work along the shared displacement interval. Panels **A–C** show the rolling 100 ps mean absolute force for CLEC-2, repaired 2GI7 and 7NMU, respectively, and panels **D–F** show the corresponding signed actuator work integrated from the beginning of loading. Each curve represents one of five independent starting configurations. The horizontal coordinate is prescribed restraint travel, and the same force and work scales are used for all three models.

**Table 1:** Matched first 5 nm restraint travel. Values are medians with first and third quartiles from five independent starts per model.

Model	Force (pN)	Work (kJ/mol)
CLEC-2	674.6 [610.8, 820.1]	191.2 [185.5, 287.1]
Repaired 2GI7	354.6 [307.8, 354.9]	148.9 [124.0, 149.6]
7NMU	576.8 [553.5, 627.0]	783.1 [765.9, 904.2]

**Fig 5:**
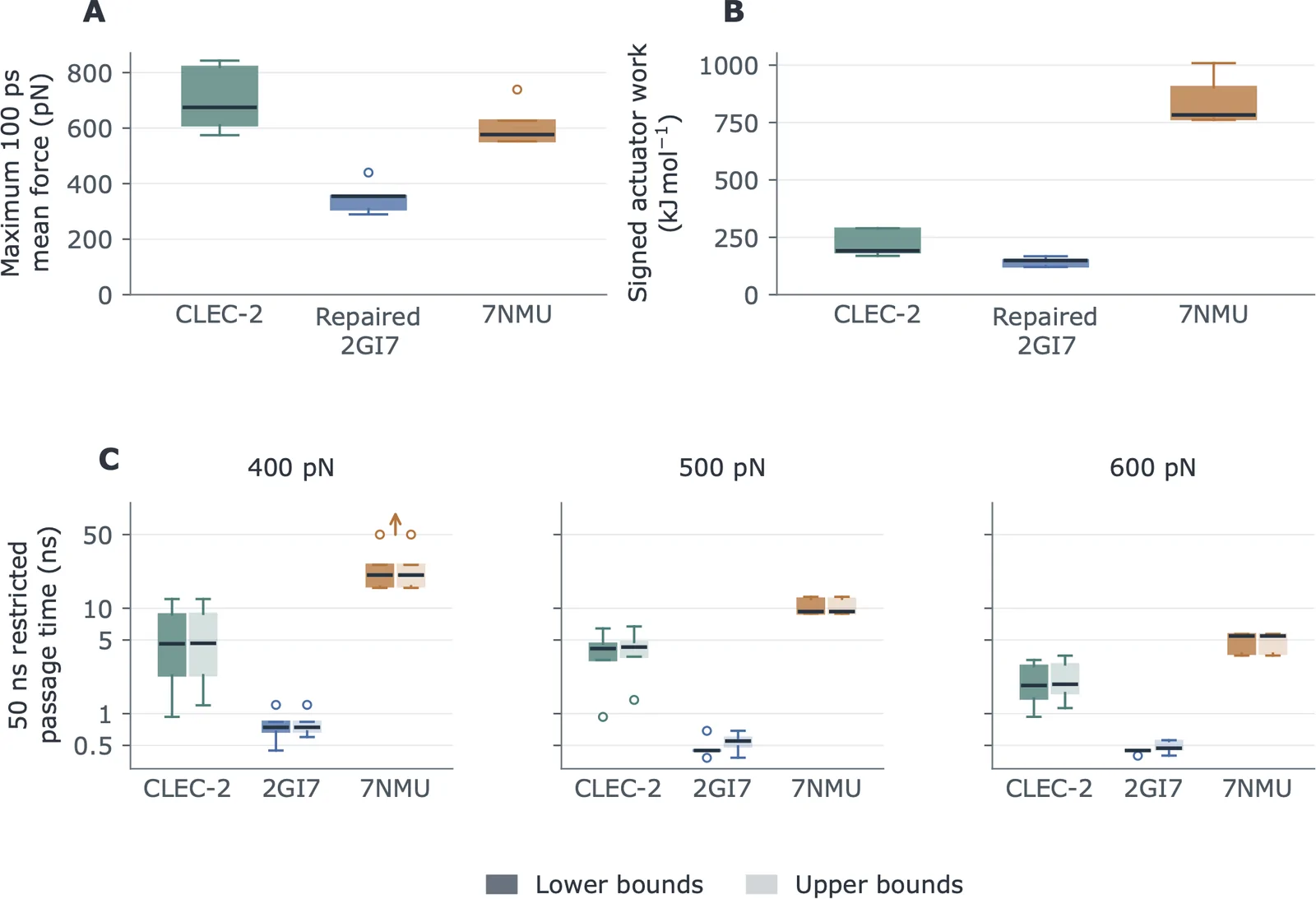
Comparison under matched loading conditions. Panels **A and B** show the maximum 100 ps mean force and signed actuator work over the initial 5 nm of restraint travel for five independent starts per model. Boxes show interquartile ranges with medians and 1.5-IQR whiskers. Panel **C** compares lower- and upper-bound passage-time assignments at each force on a logarithmic axis. The upward arrow marks the censored 7NMU passage time beyond 50 ns. Teal, blue, and orange denote CLEC-2, repaired 2GI7, and 7NMU, respectively.

Figure 5B shows median signed actuator works of 191.2, 148.9, and 783.1 kJ mol^–1^ for CLEC-2, repaired 2GI7, and 7NMU, respectively. Work was highest for 7NMU and lowest for repaired 2GI7, with *p* = 0.0476 for all pairwise comparisons. By 2 nm of restraint travel, CLEC-2 and repaired 2GI7 had accumulated most of their total work, whereas 7NMU had accumulated only 34.2%, consistent with its resistance extending to later displacement and its much greater total work.

Because interreceptor contacts persisted throughout the matched displacement interval, additional longer-displacement trajectories were examined to determine whether the receptor pairs would eventually separate. All five repaired 2GI7 trajectories completed 100 ns and reached approximately 10 nm of directional separation, with no interchain heavy-atom contacts within 0.6 nm at the endpoints. Endpoint monomer Cα RMSDs remained 0.194–0.452 nm, with peptide and disulfide geometries preserved. In contrast, four 7NMU trajectories reached 100 ns and one approximately 96 ns, while late-time configurations retained interreceptor contacts. Extended loading therefore produced complete separation of repaired 2GI7, whereas 7NMU retained residual association. A representative final snapshot of 7NMU (Fig 6) shows substantial extension of the receptor pair while a localized interchain contact remains, consistent with the nonzero contacts observed in the late-time analysis.

**Fig 6:**
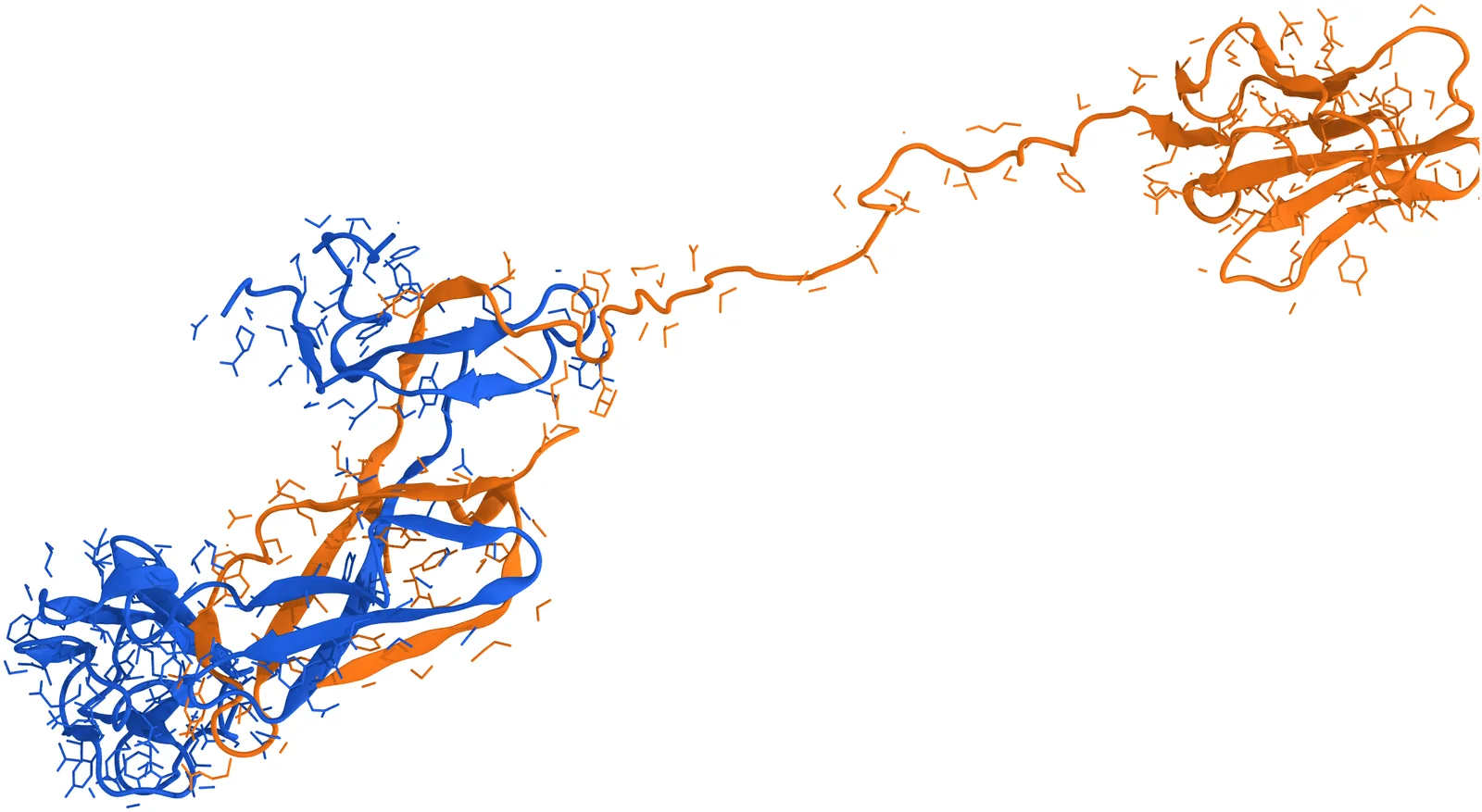
Representative final snapshot of the extended 7NMU trajectory. Despite substantial displacement, localized contact between the two receptor chains remains.

### 3.3 Constant force

Coordinate reconstruction recovered all fifteen repaired GPVI passages to a 10 nm increase. Two crossings were resolved from directly recorded dense coordinate samples and thirteen from calibrated pulling-potential observations, with reconstruction errors below $7\times 10^{-5}$ nm over the calibration interval. Figure 5C compares all three preparations at the common 5 nm endpoint.

Repaired GPVI showed the shortest passage times, with median intervals of 0.741–0.742, 0.447–0.550, and 0.447–0.470 ns at 400, 500, and 600 pN, respectively. The corresponding CLEC-2 medians were 4.600–4.650, 4.143–4.280, and 1.852–1.900 ns, whereas 7NMU gave 20.651, 9.307, and 5.459 ns. Repaired GPVI remained separated from 7NMU at all three forces (*p* = 0.0238 for each comparison). The upward arrow in Fig 5C marks the 7NMU trajectory censored at 50 ns under 400 pN.

### 3.4 Effect of the repaired 2GI7 model

The original 2GI7 model (cohort II) and the repaired model retain the same geometry for the 2719 resolved heavy atoms. Reconstruction adds 17 missing residues in chain B at positions 99–107 and 130–137, increasing the dimer from 349 to 366 modeled residues and converting four protein fragments into two continuous chains. Figure 7 shows the repaired regions, while Fig 8 compares the two simulation ensembles.

**Fig 7:**
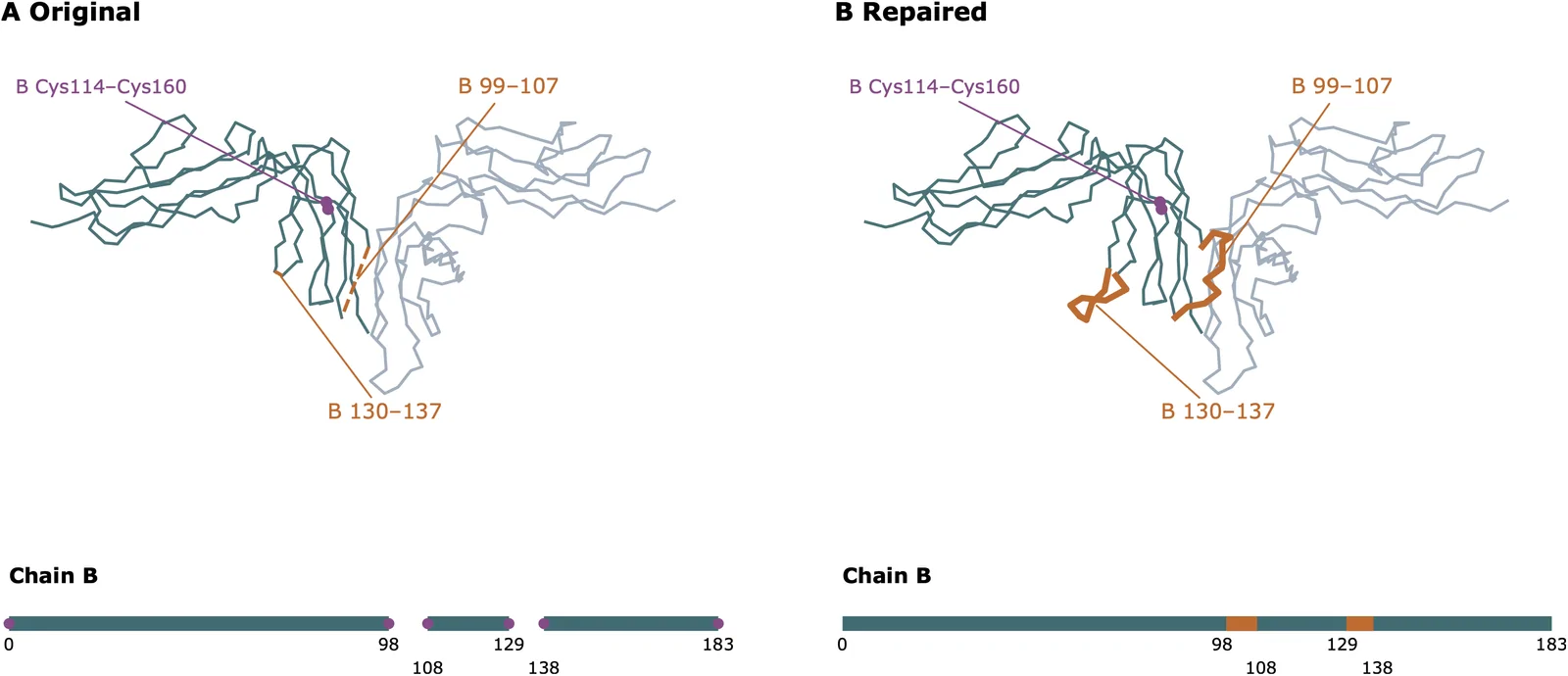
Completion of chain B in 2GI7. Panels **A and B** show actual original and reconstructed input coordinates in the same Cα projection, with chain A in gray and chain B in teal. Orange traces locate the modeled segments at residues 99–107 and 130–137, and purple markers locate the Cys114–Cys160 sulfur pair. Dashed gap connectors mark the unresolved segments in the earlier preparation. The lower sequence diagrams show the three original chain B fragments and the continuous reconstructed chain. The sulfur pair is linked by a disulfide in the reconstructed molecular topology.

**Fig 8:**
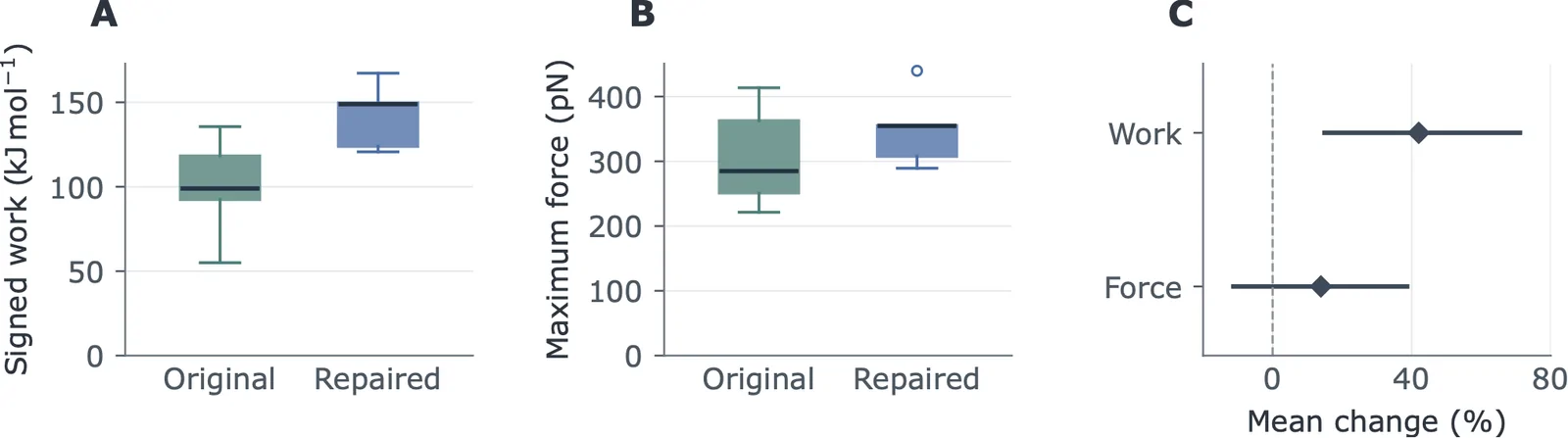
Sensitivity to 2GI7 structural preparation. Panels **A and B** compare signed actuator work and maximum 100 ps mean force over a 5 nm restraint displacement for five starts per preparation. Boxes show the interquartile ranges and medians, with whiskers extending to the non-outlier observations. Original and repaired 2GI7 are shown in teal and blue.

At the common 5 nm restraint displacement, reconstruction increased the mean work from 100.05 to 142.09 kJ mol^–1^, a difference of 42.04 kJ mol^–1^. The corresponding *p* value was 0.0635 after adjustment. The mean maximum force increased more modestly, from 306.72 to 349.30 pN, with substantial overlap between the two distributions (*p* = 0.3492). Neither difference reached statistical significance after adjustment.

Despite these changes, both 2GI7 preparations retained lower median force and work than CLEC-2 over the same loading interval. Under constant force, the repaired 2GI7 model also showed slightly longer passage times, with mean differences relative to the original preparation of 0.039–0.271 ns at 400 pN, 0.016–0.275 ns at 500 pN, and 0.060–0.315 ns at 600 pN. Overall, reconstruction produced only modest changes in passage time and did not alter the comparative mechanical behavior of 2GI7.

### 3.5 Force-dependent passage times

Table 2 distinguishes the restricted and exponential mean passage times, and Fig 9 shows the survival bounds obtained from the individual passage intervals, with all three models evaluated at the same 5 nm extension and 50 ns observation horizon. The area under these curves gives restricted mean times of 0.783–0.814, 0.482–0.540 and 0.437–0.488 ns for repaired 2GI7 at 400, 500 and 600 pN, respectively, compared with 25.642–25.643, 10.451–10.452 and 4.815–4.816 ns for 7NMU.

**Table 2:** Mean passage times at the common 5 nm endpoint. The restricted means integrate survival through 50 ns and retain event-time bounds. The exponential means additionally assume constant hazard at each force.

Model	Force (pN)	Restricted mean (ns)	Exponential mean (ns)
CLEC-2	400	5.759–5.850	5.804
CLEC-2	500	3.867–4.120	3.992
CLEC-2	600	2.049–2.216	2.131
Repaired 2GI7	400	0.783–0.814	0.798
Repaired 2GI7	500	0.482–0.540	0.510
Repaired 2GI7	600	0.437–0.488	0.462
7NMU	400	25.642–25.643	32.053
7NMU	500	10.451–10.452	10.451
7NMU	600	4.815–4.816	4.816

**Fig 9:**
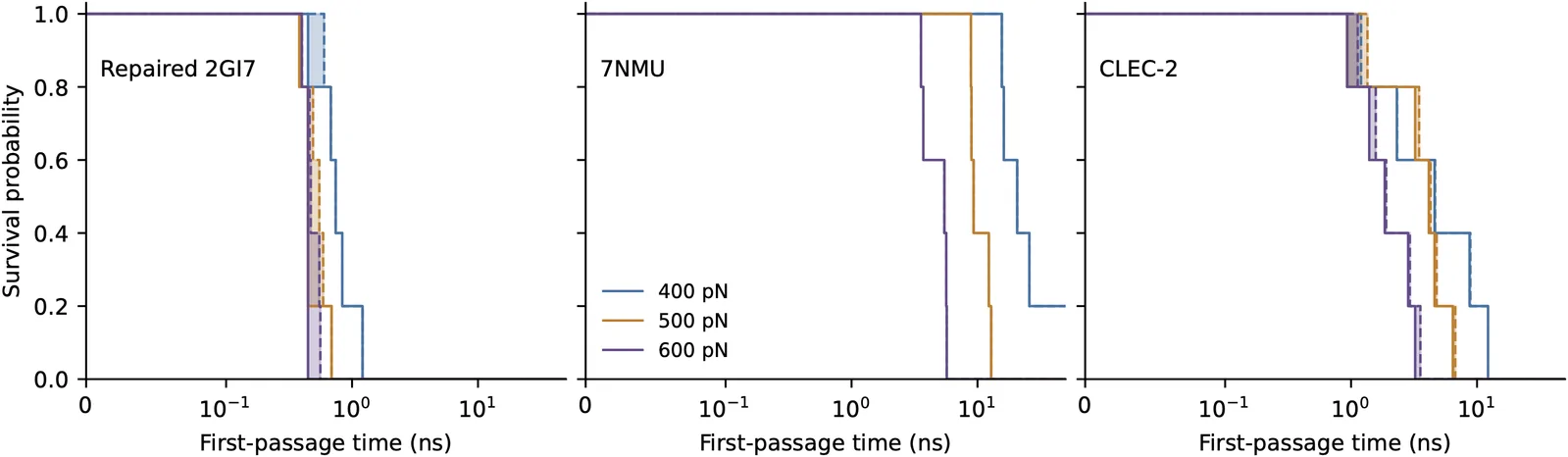
Survival of the common directional extension endpoint. The curves retain the intervals containing each observed passage and the observation censored at 50 ns. Solid and dashed curves bound the empirical survival function, and shading between them shows temporal identification uncertainty. The horizontal scale is linear near zero and logarithmic above 0.1 ns, with a common 50 ns endpoint. Each model contains five independent starting configurations at each force.

The corresponding CLEC-2 restricted means are 5.759–5.850, 3.867–4.120 and 2.049–2.216 ns, placing its survival area between those of the two GPVI preparations at each force. These ranges retain the earliest and latest event assignments consistent with the temporal observations, while the one 7NMU trajectory censored at 400 pN contributes its entire 50 ns observation period to the restricted mean.

Under the exponential distribution, separate estimates at each force give mean passage times of 0.798, 0.510 and 0.462 ns for repaired 2GI7, 32.053, 10.451 and 4.816 ns for 7NMU, and 5.804, 3.992 and 2.131 ns for CLEC-2. The larger 400 pN estimate for 7NMU relative to its restricted mean follows from the exponential continuation of the survival curve beyond the finite observation horizon, making the dependence on the distributional assumption explicit in the comparison shown in Fig 10.

**Fig 10:**
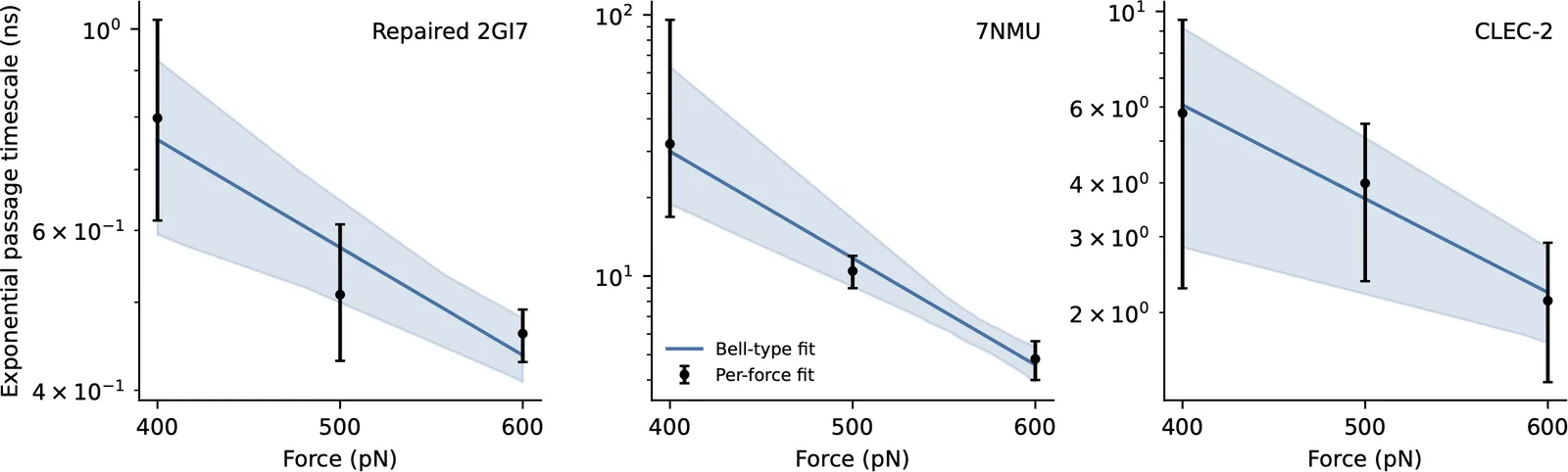
Force dependence of the geometric passage timescale under an exponential distribution. Points show separate fits at each force, lines show the Bell relation, and the bars and shaded regions show 2.5th–97.5th percentile ranges from resampling starting configurations. Vertical scales differ between models and are logarithmic. The fits cover the three applied forces and summarize the passage to a 5 nm increase in directional separation.

The Bell relation summarized the decline in mean passage time with force, with dimensionless slopes per 100 pN of 0.273 for repaired 2GI7, 0.941 for 7NMU and 0.501 for CLEC-2. Grouped resampling of the five starting configurations gave slope ranges of 0.174–0.378 for repaired 2GI7, 0.681–1.348 for 7NMU, and 0.251–0.603 for CLEC-2. The corresponding fitted passage times at 500 pN were 0.575, 11.714, and 3.675 ns, respectively, showing distinct force-dependent mechanical persistence across the three preparations.

For repaired 2GI7, the fitted dimensionless intercept and slope are *a* = 0.554044 and *b* = 0.273371, giving


$$\begin{array}{c} \tau_{0}=(1\;ns)\exp[-0.554044+5(0.273371)]=2.25\;ns. \end{array}$$ (4)

For CLEC-2, a=−1.301642 and *b* = 0.500915, giving


$$\begin{array}{c} \tau_{0}=(1\;ns)\exp[1.301642+5(0.500915)]=44.98\;ns. \end{array}$$ (5)

Table 3 reports both estimates and their multiplicity-weighted intervals alongside the fitted 500 pN times. The weighted log-scale limits are 0.198110–1.419375 for repaired 2GI7 and 2.044270–4.596247 for CLEC-2, which become 1.22–4.13 ns and 7.72–99.11 ns after exponentiation. These extrapolated geometric passage times remain on the nanosecond scale and are not predictions of cellular association lifetimes measured in seconds. Their uncertainty intervals describe resampling under the assumed rate law rather than independent evidence that the same force dependence holds between zero and 400 pN.

**Table 3:** Bell passage estimates for repaired 2GI7 and CLEC-2. Parentheses give multiplicity-weighted 95% percentile intervals from resampling starting configurations. The zero force column is an extrapolation of the 5 nm geometric endpoint beyond the measured 400–600 pN interval, not a cellular association lifetime.

Model	*b* per 100 pN	τ(500pN) (ns)	$\tau_{0}$ (ns)
Repaired 2GI7	0.273 (0.174–0.378)	0.575 (0.500–0.648)	2.25 (1.22–4.13)
CLEC-2	0.501 (0.251–0.603)	3.675 (2.207–5.094)	44.98 (7.72–99.11)

## 4 Discussion

### 4.1 Mechanical differences between the preparations

Over the same restraint displacement, repaired 2GI7 showed lower force and work than CLEC-2, while comparison with the original 2GI7 preparation showed a higher mean work after reconstruction. These results reflect different aspects of the response, with the relative ordering between receptor preparations preserved despite the shift toward higher work in the repaired 2GI7 ensemble. Under extended loading, repaired 2GI7 ultimately reached complete separation, with no interreceptor heavy-atom contacts detected by either intended-pair or minimum-image measurements, while peptide and disulfide distances remained consistent with preserved local chain geometry.

7NMU accumulated more work and reached the common threshold later than repaired 2GI7, showing sustained resistance during moving-restraint loading and longer passage times under constant force. Its domain-swapped arrangement and four covalent units differ from the two continuous chains of repaired 2GI7, and close all-atom contacts persisted during the longer displacement simulations [4]. The contrasting responses of 2GI7 and 7NMU show that uncertainty in GPVI extracellular architecture can translate into order-of-magnitude differences in mechanical persistence under the same loading conditions.

### 4.2 Mechanical response and structural separation

Maximum mean force and accumulated mechanical work describe different features of the loading response. A localized resistance can produce a high force peak, whereas continued deformation and persistent interactions can accumulate work over a longer displacement. This distinction is evident for CLEC-2 and 7NMU, where the ordering of median force maxima differs from that of median work. Considering both quantities therefore provides complementary information on peak resistance and the total mechanical response over the matched loading interval.

Directional threshold passage measures how long the loaded receptor groups take to reach a defined extension, while contact distances and aligned structures describe the associated molecular configuration. For repaired GPVI, longer displacement led to loss of interreceptor contacts while local covalent geometry remained preserved. In contrast, extended 7NMU trajectories retained close atom pairs between the loaded groups. These structural measurements therefore complement the loading coordinate by distinguishing overall receptor displacement from the persistence of local interactions.

### 4.3 Biological scope

GPVI association contributes to ligand recognition in a cellular environment that also includes receptor mobility, membrane anchoring, and multivalent interactions, all of which can influence how extracellular receptor molecules encounter ligands and one another [2,3,25]. The present calculations instead begin from pre-associated extracellular configurations and examine their response to a defined loading geometry. They therefore address the mechanical behavior of specific receptor arrangements after association has formed, relating extracellular architecture to resistance, deformation, and separation along a controlled molecular pathway.

Association measurements in low-expression Syk-negative CHO-K1 cells describe ligand-mediated receptor persistence on a cellular timescale rather than passage to a defined extracellular displacement [10]. The longer persistence of CLEC-2 than GPVI associations observed in those experiments is qualitatively consistent with the shorter passage times of repaired 2GI7 relative to CLEC-2 in the present simulations, but it does not identify the extracellular GPVI arrangement present in the cellular associations. In particular, the multivalent GPVI ligands used in those experiments were derived from Nb2, which is also present in the 7NMU crystal structure, yet the 7NMU-derived preparation in our calculations shows substantially greater mechanical persistence than repaired 2GI7 and CLEC-2. This difference indicates that the cellular lifetime ordering cannot be used to assign the observed GPVI associations directly to either crystal-derived arrangement. Instead, our results show that different GPVI extracellular arrangements can have markedly different mechanical responses. Thus, the receptor geometry present within a ligand-mediated association may influence how readily that association responds to separating forces.

CLEC-2 provides a comparison with a related platelet receptor that has a different extracellular architecture. Using the same restraint stiffness, velocity, and displacement allows direct comparison of force and work responses across the GPVI and CLEC-2 preparations. The longer GPVI trajectories further examine whether local contacts persist over an extended loading interval. Because the mechanical response depends strongly on the specific molecular arrangement being loaded, receptor identity alone is insufficient to define the mechanical behavior of an extracellular association.

### 4.4 Limitations

The repaired 2GI7 structure includes modeled loop conformations, and alternative reconstructions could produce somewhat different local responses. In addition, the mechanical passage times measured here describe motion to a defined extension and should not be interpreted as irreversible dissociation times or direct cellular association lifetimes. The simulations also omit the membrane environment, complete receptor context, ligand multivalency, and intracellular components present in cells. Despite these simplifications, the in silico comparison qualitatively reproduces the experimental observation that CLEC-2 associations persist longer than GPVI associations, supporting the relevance of extracellular receptor architecture to their distinct mechanical behavior.

## 5 Conclusions

The three receptor preparations show distinct mechanical responses under matched loading, with repaired 2GI7 separating more readily, CLEC-2 showing intermediate persistence, and domain-swapped 7NMU maintaining resistance and residual contacts over extended loading. These differences show that extracellular receptor architecture and chain connectivity strongly influence how platelet receptors resist and accommodate mechanical separation.

The results further suggest that GPVI mechanical behavior cannot be assigned from receptor identity alone, because different GPVI extracellular arrangements display markedly different persistence under force. Such differences in mechanical behavior may influence how receptor associations are maintained in the dynamic environment of platelet adhesion and signaling. Together with the qualitative agreement between repaired 2GI7 and CLEC-2 and the corresponding cellular persistence measurements, the simulations support a role for extracellular receptor organization in shaping the mechanical stability of platelet receptor assemblies.
